# Decoding predicted musical notes from omitted stimulus potentials

**DOI:** 10.1038/s41598-024-61989-1

**Published:** 2024-05-15

**Authors:** Kai Ishida, Tomomi Ishida, Hiroshi Nittono

**Affiliations:** 1 Graduate School of Human Sciences, Osaka University, 1-2 Yamadaoka, Suita, Osaka 565-0871 Japan; 2 Japan Society for the Promotion of Science, Tokyo, Japan

**Keywords:** Neuroscience, Psychology

## Abstract

Electrophysiological studies have investigated predictive processing in music by examining event-related potentials (ERPs) elicited by the violation of musical expectations. While several studies have reported that the predictability of stimuli can modulate the amplitude of ERPs, it is unclear how specific the representation of the expected note is. The present study addressed this issue by recording the omitted stimulus potentials (OSPs) to avoid contamination of bottom-up sensory processing with top-down predictive processing. Decoding of the omitted content was attempted using a support vector machine, which is a type of machine learning. ERP responses to the omission of four target notes (E, F, A, and C) at the same position in familiar and unfamiliar melodies were recorded from 25 participants. The results showed that the omission N1 were larger in the familiar melody condition than in the unfamiliar melody condition. The decoding accuracy of the four omitted notes was significantly higher in the familiar melody condition than in the unfamiliar melody condition. These results suggest that the OSPs contain discriminable predictive information, and the higher the predictability, the more the specific representation of the expected note is generated.

## Introduction

Behavioral^1–4^ and neuroscientific^5–8^ studies have shown that the human brain predicts incoming sounds when listening to music^9–11^. In particular, previous studies have provided empirical evidence of expectation in the dimensions of tonality^2,6,12^ and meter^13,14^. Veridical expectations also arise from familiarity or memory of the specific musical tune^15,16^. However, it is unclear whether the prediction can be specific (“the note”) or whether it is only vague (“some notes”).

According to the predictive coding framework^17^, the size of the prediction error (i.e., the difference between the predicted input and the actual input) can be modulated by predictability^18^. In the auditory domain, event-related potential (ERP) components, such as the N1^19^ and the mismatch negativity (MMN^20–22^), have been used as prediction error signals. For example, Hsu et al.^23^ demonstrated that compared with the N1 elicited by the final tone that followed the ascending patterns, the N1 amplitude was enhanced when the ascending tone pattern was violated by a lower final tone (mispredicted), whereas the N1 amplitude was attenuated when the tone pattern before the final tone was randomly scrambled (unpredicted). However, interpretation of the ERP amplitude elicited by deviant tones is difficult because the ERP amplitude changes not only with expectation violation but also with physical parameters of the tones^19^. Instead of presenting unexpected tones, the present study used unexpected omissions and aimed to investigate whether the predictability of notes was reflected in ERPs elicited by an omission in a specific musical context.

By recording ERP responses to the omission of a sound, it is possible to avoid confounding sensory-evoked potentials with prediction error signals. For example, neural omission responses (hereafter, omitted stimulus potentials: OSPs), such as omission N1 (oN1)^24–27^ and omission MMN (oMMN)^28–30^, have been observed when a sound that is predictable in timing and content is unexpectedly omitted. They are considered a prediction error signal. The oN1 has been reported as a neural response to the omission of auditory stimuli generated by the participant’s button press or the omission of a sound that is usually presented with a visual stimulus^24–27^. Moreover, some studies have observed an N1-like response to the omission of an auditory stimulus that was not associated with a button press or visual cue^31,32^. In the case of omission, since no external stimuli are presented and bottom-up input is absent, the omission response is considered a pure reflection of top-down predictive information^25^. Furthermore, previous studies have shown that the amplitude of oN1 is larger when an omission occurs in a context where the content of the sound is predictable than when it is unpredictable ^25,27,33^. Therefore, OSPs would be a better indicator of the predictive process in music than ERPs elicited by deviant tones.

The present study investigated whether the predictability of the specific content of an upcoming sound affects the omission-related neural response during passive music listening. Familiar melodies of well-known Japanese songs and newly created unfamiliar melodies were used for predictable and unpredictable conditions, respectively. In both types of melodies, four types of notes (E, F, A, and C) were presented or omitted with equal probability at the same position in each melody. Even without the button presses or visual cues often used in previous OSP research, the sense of beat and meter in music will help the listener predict the exact timing of the notes to be presented in the present study. If the OSPs are modulated by specific predictive information about the content of the note, the amplitude of oN1 will be larger in the familiar melody condition than in the unfamiliar melody condition, reflecting the predictability of the note.

To provide further evidence that the OSPs contain specific information about the content of the predicted but omitted note, the present study attempted to decode the identity of the omitted note from them. Support vector machine (SVM), a type of supervised machine learning, was used for decoding, because Trammel et al.^34^ demonstrated that SVM performed better than other machine learning methods, such as linear discriminant analysis and random forest, in decoding ERPs elicited by related or unrelated words. Using the SVM, Bae and Luck^35^ decoded 16 directions of stimuli from ERP responses recorded from participants who performed a direction judgment task for a visual stimulus (teardrop shape). Furthermore, Salehzadeh et al.^36^ decoded 12 categories of finger-numerical configurations (i.e., positioning fingers in relation to numerical concepts or counting) from ERP responses. In line with these studies, the present study attempted to decode the four pitch categories of omitted notes from OSPs. If the predictive information about the identity of the note was contained in the OSP, the decoding accuracy would be higher in the familiar melody condition than in the unfamiliar melody condition.

## Results

**Figure 1:**
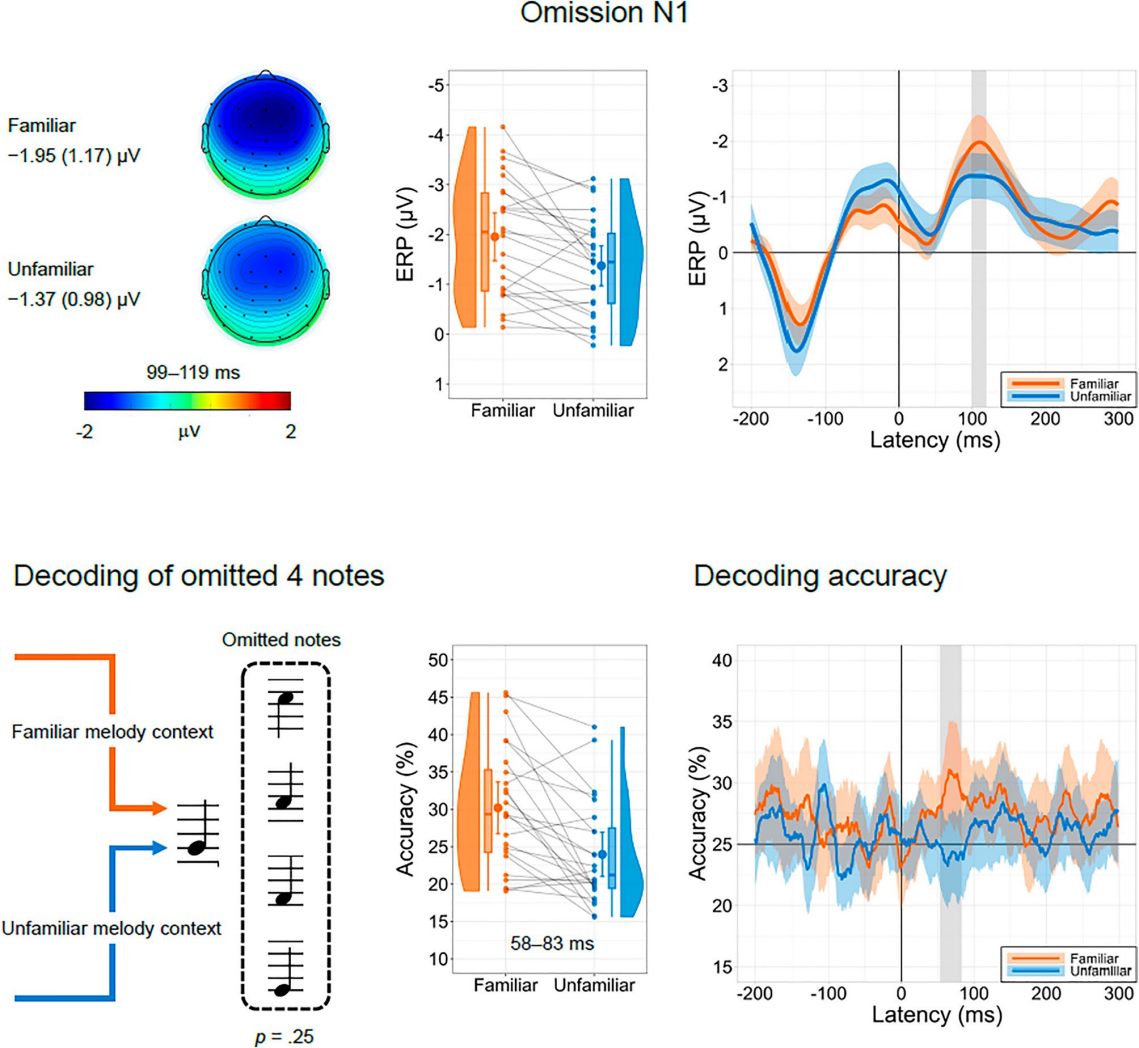
Omitted stimulus potentials and decoding accuracy. The upper panel shows the ERP waveforms (means of the four frontocentral electrodes: Fz, FC1, FC2, and Cz) with 95% confidence intervals (CIs) during the omission of notes in the familiar and unfamiliar melody conditions. The scalp topographies and mean (*SD*) amplitudes of the oN1 response (99–119 ms) are also shown. The lower left panel shows the four omitted notes. The lower right panel shows the decoding accuracy with 95% CI in the whole period and the mean accuracy in the 58–83 ms interval. Large dots and error bars in the raincloud plots indicate the mean ERP amplitudes and accuracy across participants and their 95% CIs.

Figure 1 shows the OSP waveforms and the accuracy of decoding the identity of the omitted notes. The oN1 amplitude was calculated as the mean amplitude of 99–119 ms interval. The oN1 amplitude was significantly larger in the familiar melody condition (*M* =  − 1.95 μV, *SD* = 1.17 μV) than in the unfamiliar melody condition (*M* =  − 1.37 μV, *SD* = 0.98 μV), *t*(24) =  − 3.67, *p* = 0.001, *dz* =  − 0.73, BF_10_ = 29.41.

Decoding accuracy was higher in the familiar melody condition than in the unfamiliar melody condition. The cluster-based permutation test revealed a significant difference in the decoding accuracy between the familiar and unfamiliar melody conditions in the 58–83 ms interval, *t*_*sum*_ = 87.11, *p* < 0.001. The mean accuracy of this interval was above chance level in the familiar melody condition (*M* = 30.2%, *SD* = 8.2), *t*(23) = 3.10, *p* = 0.005, *dz* = 0.63, BF_10_ = 8.67, but not in the unfamiliar melody condition (*M* = 24.0%, *SD* = 7.0), *t*(23) =  − 0.72, *p* = 0.479, *dz* =  − 0.15, BF_10_ = 0.27.

## Discussion

The present study investigated the specificity of the predictive information generated according to the predictability of omitted notes in the musical context. The predictability of the notes was manipulated by melody familiarity, and sensory-evoked responses were eliminated by recording OSPs elicited by omitted target notes. Consistent with the predictive coding framework, unexpected omissions in the familiar melody condition elicited larger oN1 response than those in the unfamiliar melody condition. These results suggest that the predictability of the omitted notes was reflected in the OSPs during passive listening. Moreover, the decoding accuracy of the omitted notes was significantly higher in the familiar melody condition than in the unfamiliar melody condition. Thus, the present study suggests that the more predictable the notes, the more specific the predictive representation.

Previous studies have reported that a larger oN1 response occurs in predictable contexts than in unpredictable contexts^25,27,33^. The larger prediction error response to deviance in the familiar context than in the unfamiliar context is also observed in previous MMN research^37,38^. This larger neural response is consistent with the concept of precision-weighted prediction error^39–43^. Precision is the inverse of the variance of a (probabilistic) distribution and reflects certainty about a variable such as sensory input^41,42^. The higher the precision (i.e., high predictability and low uncertainty), the higher the sensitivity and the higher the gain of sensory input^39,40^. In the present study, the uncertainty of familiar melodies was lower than that of unfamiliar melodies because the memory representation of the melody facilitates the generation of the prediction. Thus, the occurrence of the unexpected omission may be more salient in the familiar melody than in the unfamiliar melody, where the occurrence of the note is ambiguous, and this would result in a larger oN1 response in the familiar melody condition.

Another possibility is that the repetitions of the familiar melody may have induced stronger anticipation and attention to the target position than the unfamiliar melody. Attention optimizes the precision of prediction errors^41^, and the facilitation of top-down and attentional processing in the familiar melody condition could enhance the oN1 response. It is also noteworthy that the oN1 in the present study had a frontocentral distribution, which differs from the typical oN1 observed predominantly at temporal electrodes^25,26,33^. Janata^31^ reported that a similar frontocentral N1-like potential was elicited by the omission of a tone that participants actively anticipated. It is possible that a strong top-down processing to the target position elicits a different negativity in the oN1 time window.

The oN1 was elicited even in the unfamiliar melody condition. This may be because the expectation that any melody would continue was violated by the omission. Dercksen et al.^33^ reported that the oN1 occurred when the timing of a stimulus was predictable but its content was not. In the present study, consistent with their findings, omissions in the unfamiliar melody condition elicited an oN1 when the omission occurred in a continuous melodic context. This temporal prediction should be inherent in musical materials. A sense of beat and rhythm may facilitate better temporal prediction and reduce latency jitters, which may prevent the stable recording of early OSPs^44^. Thus, the present study shows that oN1 occurs even in the “I don’t know what the upcoming stimulus is, but some stimulus is coming in this time sequence” situation by using an ecologically valid stimulus with clear timing information.

The accuracy of decoding the omitted note identity was higher in the familiar melody condition than in the unfamiliar melody condition. The significant differences were found in an early latency range (58–83 ms). While the amplitude of oN1 reflect the predictability of melody notes, they do not directly reflect the specificity and clarity of the prediction of note identity based on familiarity. The SVM decoding of the omitted notes from OSPs allows for a more direct examination of the prediction of note identity content compared to examining ERP amplitudes. The results support the notion that the OSPs reflect predictive signals containing specific information about the upcoming stimulus^45^. In an MEG study, Demarchi et al.^46^ showed that decoding accuracy of the feature (carrier frequency) of omitted sounds increased as the sound sequence became more predictable (i.e., arranged according to probabilistic rules). Our results extend their finding to more complex and realistic musical stimuli. SanMiguel et al.^26^ suggested that prediction inducted a sufficient sensory template for the expected sound, at least up to the oN1 latency range (56–112 ms). Bendixen et al.^47^ also suggested that the brain is set up to process the expected tone by default and only interrupts processing when an omission is detected. The fact that the latency range with high decoding accuracy (i.e., 58–83 ms) was different from the latency range of oN1 may reflect that the predictive representation was strongly retained before the omission was detected and the prediction error was elicited. These results suggest that when the predictability of the musical context is high, ERP responses during omissions contain information about more specific pitch expectations, at least immediately after the onset of the omission.

The current results should be interpreted carefully. First, although the present study focused on melody familiarity (i.e., note arrangement), it also includes rhythmic and harmonic dimensions. Even though the familiar and unfamiliar melodies were similar in terms of beat, tempo, and number of notes, not only the melodic but also the harmonic and rhythmic predictions of the upcoming notes may differ between the two conditions. Second, the preceding notes before the target position were not rigorously controlled, except that the immediately preceding tone was set to G. That is, the possibility that preceding acoustic and structural differences affected the oN1 time window cannot be ruled out. However, our decoding analysis suggests that the OSP in the familiar melody condition contained more concrete expectation information for the omitted notes than the OSP in the unfamiliar melody condition. Even though ERP waveform differences might reflect previous acoustic and structural differences, we can say that the brain electrical signals of this time window contain predictive information. To address these issues, future research should use a more rigorous manipulation of familiarity, such as creating two new melodies that are equivalent in harmony and rhythm, and having participants learn one melody and leave the other unheard. Third, the current protocol was not typical for the oN1 recording because the omitted stimulus was not associated with a button press or visual cue. Although the listener can predict the timing of notes from the sense of beat and meter in music, this difference in protocol may have resulted in an atypical frontocentral oN1, which differs from the temporal oN1 reported in previous studies^24–26,33^. Finally, the time intervals of the clusters identified by the cluster-based permutation test do not necessarily indicate the onset and offset points of the effects^48^. Further research is needed to determine whether the familiarity effect on decoding accuracy is observed only before the elicitation of prediction errors or whether it also occurs in the oN1 time window.

In conclusion, the present study demonstrated that unexpected omissions in the familiar melody condition elicited a larger oN1 than unexpected omissions in the unfamiliar melody condition. These findings suggest that the oN1 reflect the predictability of the pitch in melody based on the melody’s familiarity. Moreover, the SVM successfully classified the identity of omitted notes, and the decoding accuracy was higher in the familiar melody condition than in the unfamiliar melody condition. These results provide evidence that the ERP during the omission contains distinguishable predictive information, and the higher the predictability, the more the specific representation of the expected note is contained.

## Methods

### Ethics

The protocol of the present study was approved by the Behavioral Research Ethics Committee of the Osaka University School of Human Sciences, Japan (HB023-075) in accordance with the Declaration of Helsinki. Written informed consent was obtained from all participants. Participants received a cash voucher of 2500 Japanese yen as an honorarium.

### Participants

A sample size of 21 was predetermined to ensure the detection of a medium effect size (*d*_z_ = 0.66) for the difference in the oN1 amplitude between the high and low predictability. This was calculated using data from SanMiguel et al.^25^ and reported in Dercksen et al.^33^, with power 1 − β = 0.80 and error rate α = 0.05. The calculation was performed using G*power^49^. Taking into account data exclusions, 25 participants were recruited. They were all nonmusicians who had never worked with music professionally. Finally, data from all 25 participants (16 women and 9 men, 19–35 years old, *M* = 22.0 years) were used for the analysis of the oN1. The decoding analysis was done with the data from 24 participants because one participant did not have a sufficient number of artifact-free trials of each note (less than 80% or 50 trials) to avoid the risk of overfitting. Twenty-three participants were right-handed, and two were left-handed (FLANDERS handedness questionnaire^50^). None reported having hearing impairments or a history of neurological disease. All participants confirmed that they knew the four familiar melodies used in the experiment. The participants’ musical ability was evaluated using the Japanese Gold-MSI questionnaire^51^ (the original is Müllensiefen et al.^52^), which evaluates General Sophistication (*M* = 62.5, *SD* = 18.0) as well as subscales of Active Engagement (*M* = 30.0, *SD* = 8.3), Perceptual Abilities (*M* = 38.7, *SD* = 10.4), Musical Training (*M* = 20.9, *SD* = 10.2), Emotions (*M* = 29.1, *SD* = 7.3), and Singing Abilities (*M* = 24.3, *SD* = 8.7). Four participants had self-reported absolute pitch.

### Stimuli and procedure

**Figure 2:**
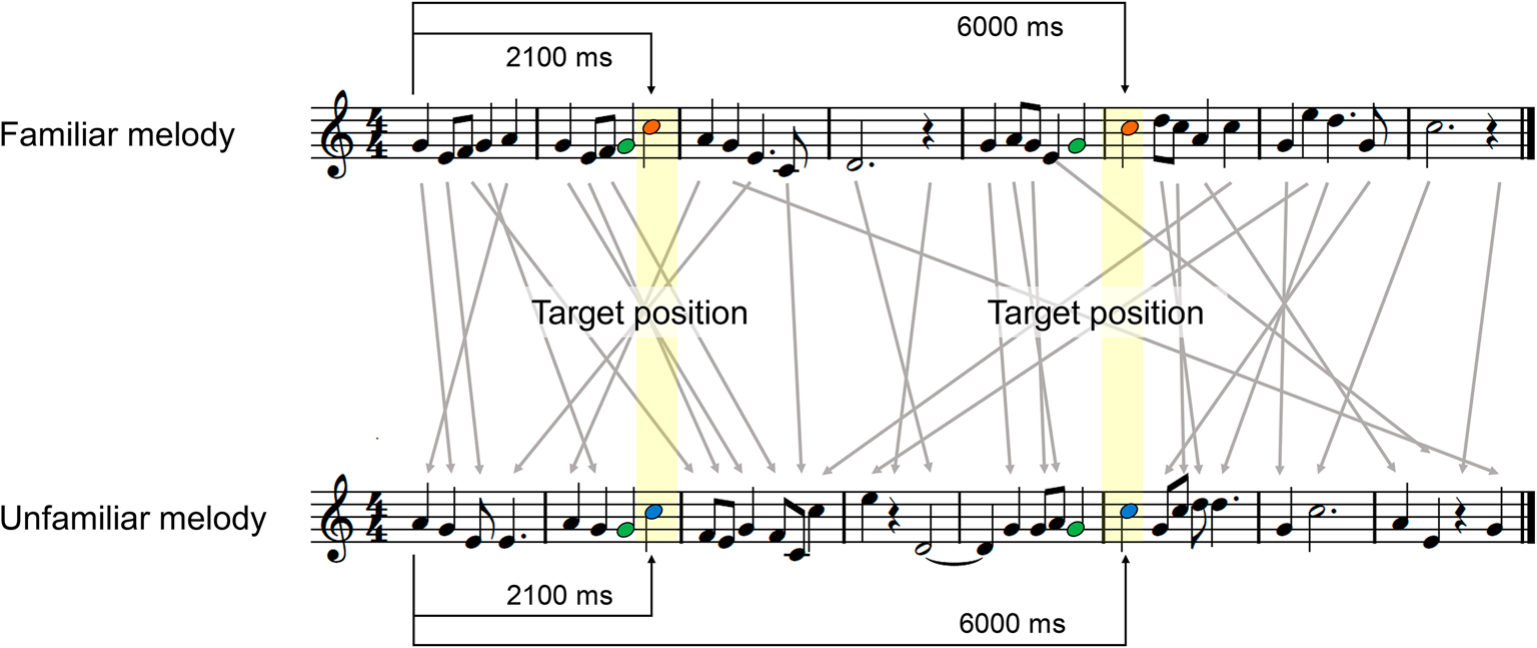
Samples of familiar and unfamiliar melodies. The upper part shows the familiar melody “Harugakita,” and the lower part shows its unfamiliar version. As indicated by the gray arrows, the unfamiliar version was created by shuffling the positions of pauses and notes of the corresponding familiar melody while keeping the positions of the target notes the same as in the original familiar melody. The tone and omission conditions (50% each) were manipulated at the positions of the orange notes in the familiar melody and the blue notes in the unfamiliar melody. In both types of melodies, the quarter notes E, F, A, and C are omitted after the quarter note G, which is colored green.

The stimuli used in this study are available at https://osf.io/4q7x6/. The sample of the stimulus is shown in Fig. 2. The familiar melodies consisted of four famous Japanese songs used as teaching materials for music education in Japan. Table 1 shows the profiles of each melody. Two melodies were in C major, the other melodies were in F and B♭ major, and all melodies were played with piano timbre. All melodies were in the 4/4 time signature. The duration of each melody was 9.6–19.2 s (200 bpm). The target quarter notes (300 ms) of E, F, A, and C following the quarter note of G were omitted with a probability of 50%. These four notes were called target notes, and their positions were called target positions. The unfamiliar melodies were created by shuffling the pause and note positions for each of the four familiar melodies, while keeping the target note positions the same as in the familiar melodies. More specifically, the unfamiliar melodies retained the same beat, tempo, and number of respective notes as the original familiar melodies, while they had different rhythmic and harmonic structures than the original melodies. All melodies were presented with an interstimulus interval of 600 ms. Although the familiar and unfamiliar melodies were different, both contained the same notes and the same target positions. Note that the same G note was presented before the omission in both types of melodies. Thus, the difference in omission responses between the familiar and unfamiliar melodies reflects the predictability of the melodies based on familiarity rather than the late ERP components elicited by the preceding note before the omission.

**Table 1:** Attributes of each familiar melody and the numbers of notes in the target positions.

		Song title				Sum
		*Momiji*	*Harugakita*	*Harunoogawa*	*Yuuyakekoyake*	
Note identity	E			6		6
	F	1			5	6
	A	2		4		6
	C	2	2	3		7
Duration (s)		19.2	9.6	19.2	19.2	67.2
Key		F major	C major	C major	B♭ major	

Prior to the EEG recording, the participants completed the FLANDERS questionnaire^50^ and the Japanese Gold-MSI^51^. The EEG recording consisted of four familiar melody blocks and four unfamiliar melody blocks. The order of the eight blocks was randomized. Four melodies were randomly presented 20 times each, resulting in a total of 60–70 presentations for each combination of G–E/F/A/C in the four blocks of familiar melodies and four blocks of unfamiliar melodies. Thus, the tone and omission conditions each consisted of 250 trials in each melody block (i.e., 60 + 60 + 60 + 70 = 250 trials). Participants were asked to ignore the melodies while watching a silent movie. Including the online questionnaire session, electrode preparation, and short breaks between blocks, the entire experiment took approximately 2.5 h.

### EEG recording

EEG data were recorded using QuickAmp (Brain Products, Germany) with Ag/AgCl electrodes. Thirty-four scalp electrodes were placed according to the 10–20 system (Fp1/2, F3/4, F7/8, Fz, FC1/2, FC5/6, FT9/10, C3/4, T7/8, Cz, CP1/2, CP5/6, TP9/10, P3/4, P7/8, Pz, O1/2, Iz, PO9/10). Additional electrodes were placed on the left and right mastoids, the left and right outer canthi of the eyes, and above and below the right eye. The data were referenced offline to the nose-tip electrode. The sampling rate was 1000 Hz. The online filter was DC-200 Hz. Electrode impedances were kept below 10 kΩ.

### EEG data reduction

EEG data were analyzed using EEGLAB (Delorme & Makeig^53^; Version 2023.1) on MATLAB R2022b (The MathWorks Inc., Natick, MA). First, a digital filter of 0.5–25 Hz was applied to the data (SanMiguel et al.^26^). A zero-phase Hamming-windowed sinc finite-impulse-response filter based on the firfilt EEGLAB plugin was used. Independent component analysis (InfoMax algorithm, which is the default of EEGLAB) was then applied to the filtered data to remove ocular, heartbeat, and muscle artifacts. Component rejection was semiautomated using the ADJUST algorithm, which automatically detects the artifact-specific spatial and temporal features^54^, and visual inspection. On average, 13.9 ICs (*SD* = 2.4) were rejected as artifacts. A period of 500 ms (200 ms before and 300 ms after the target position) was averaged after removing trials with voltages exceeding ± 80 μV at any channel. Baseline correction was applied by subtracting the mean amplitude of the 200 ms prestimulus period from each point of the waveform. For statistical analysis, the frontocentral electrodes (Fz, Cz, FC1, FC2) were clustered, and the mean ERP waveform of the electrodes was calculated. The selection of these electrodes was based on the result of a preliminary study, in which the oN1 was dominant in the frontocentral region. In the present study, the oN1 was also dominant in the frontocentral region (see Supplementary Information for ERP waveforms at other scalp regions).

The grand mean waveforms of the omissions in the familiar and unfamiliar melody conditions were averaged (averaged grand mean waveforms). Then, the peak of oN1 (109 ms in the present study) was detected in the interval of 50–110 ms, and the interval ± 10 ms (99–119 ms in the present study) from the peak was defined as the oN1 interval. The 50–110 ms interval was predetermined on the basis of previous studies (Dercksen et al.^33^: 42–92 ms; van Laahoven et al.^27^: 45–80 ms). On average, 240 (126–250) and 246 (222–250) epochs were used to calculate the oN1 amplitudes of familiar and unfamiliar melody conditions, respectively.

### Decoding

Decoding was performed using the ERPLAB Toolbox (Version 10.02)^55^. The classification method was One-vs-Rest. The decoding method used in this study was similar to that of Bae and Luck^35^, who performed a participant-based approach using the SVM. The SVM was run separately on familiar and unfamiliar omission ERP waveforms for each participant at each time point. Voltages from 34 scalp electrodes were used as feature values. Threefold cross-validation was conducted at each time point to assess the generalizability of the model. In the threefold cross-validation, all trials of each note were randomly divided into three blocks. Two of the three blocks were used for training, and the remaining block was used for testing the classifier to calculate decoding accuracy. This process was repeated three times until all three blocks were used as the test block. The averaged decoding accuracy over the three test datasets was then calculated. For each time point, threefold cross-validation was repeated 20 times (iterations), and the averaged decoding accuracy was calculated. Decoding was performed in the full range of − 200–300 ms after the onset of the omission.

### Statistical analysis

Statistical analyses were performed using JASP 0.17.1^56^. To examine the difference in oN1 amplitude between familiar and unfamiliar melody conditions, a two-tailed paired *t*-test was conducted on the mean ERP amplitude of the oN1 interval. The standardized mean difference effect size for within-participants designs, *dz,* was reported^57^. A Bayesian paired *t*-test was then performed to assess the evidence for the absence (effect size δ = 0, null hypothesis) or presence (effect size δ > 0, alternative hypothesis) of the difference. The difference in decoding accuracy of the full ERP range (− 200–300 ms) between the familiar and unfamiliar melody conditions was tested using the cluster-based permutation test^58,59^. The number of iterations was 10,000. For frequentist hypothesis testing, the significance levels were set at α = 0.05. For Bayesian hypothesis testing, the Cauchy distribution with a scale parameter *r* of 0.707 was used as the prior distribution for δ in the *t*-test. According to the classification scheme of Schönbrodt and Wagenmakers^60^, a Bayes factor (BF_01_) greater than 3 was considered moderate evidence for the null hypothesis. The stimulus materials and the data necessary to replicate the statistical results are available at https://osf.io/4q7x6/.

### Supplementary Information

Supplementary Information.

## Data Availability

The sound materials used and datasets analyzed for the present paper are available at https://osf.io/4q7x6/.
