# Supplementary material for: Decoding predicted musical notes from omitted stimulus potentials
Source: Sci Rep. 2024 May 15;14:11164. doi: 10.1038/s41598-024-61989-1 (PMC11096333; doi:10.1038/s41598-024-61989-1)
Supplement: Supplementary file 1 — Supplementary Information. [file 41598_2024_61989_MOESM1_ESM.pdf]

Supplementary Materials

oN1 Response in Other Scalp Regions

Supplementary Figure S1 shows the ERP waveforms elicited by omissions in the familiar and unfamiliar melody conditions at three scalp regions: left temporal (F7, FC5, FT9, and T7), frontocentral (Fz, FC1, FC2, and Cz; as reported in the main text), and right temporal (F8, FC6, FT10, and T8).

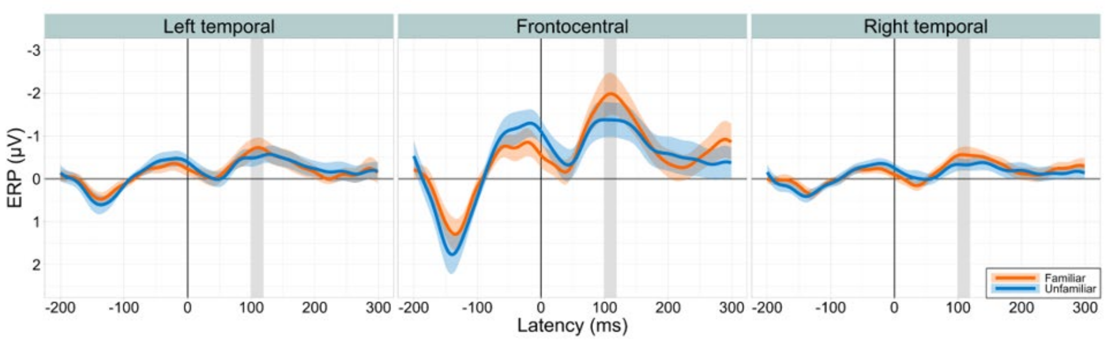

Supplementary Figure S1. oN1 response in the frontocentral and left and right temporal regions. The ERP waveforms are shown with 95% CIs.

N1 Response to Tones

Supplementary Figure S2 shows the ERP waveforms and the N1 amplitudes for the tone condition in the familiar and unfamiliar melodies. Similar to the oN1, the peak of the N1 (89 ms) was detected in the interval of 50–110 ms, and the interval ± 10 ms (i.e., 79–99 ms) from the peak was defined as the N1 interval. On average, 240 (124–250), and 246 (222–250) epochs were used to calculate the oN1 amplitude of familiar and unfamiliar melodies, respectively. The difference in the N1 amplitude between the familiar (M = −0.74, SD = 0.91) and unfamiliar (M = −0.91, SD = 1.40) melodies was not significant, $t(24) = 0.73$, $p = .471$, $dz = 0.15$, $BF_{10} = 0.27$.

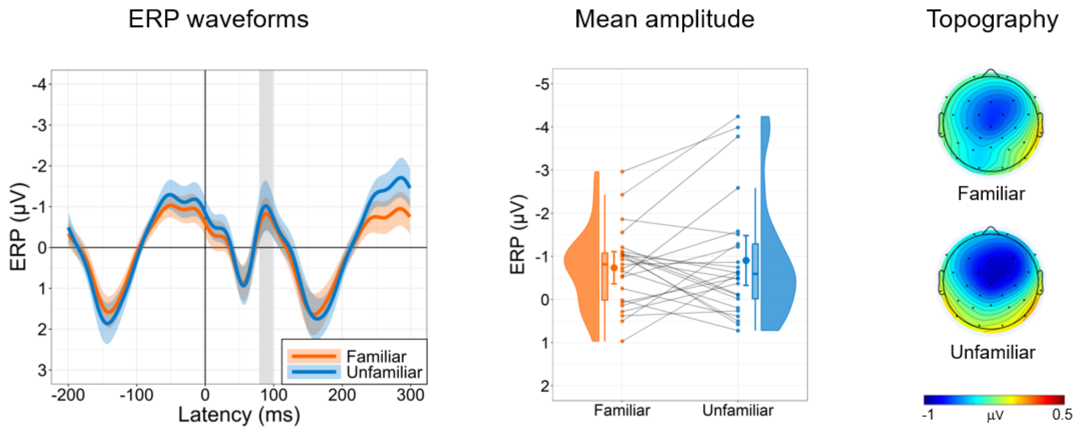

Supplementary Figure S2. N1 response to the tone condition. The left panel shows the ERP waveforms (means of the four frontocentral electrodes: Fz, FC1, FC2, and Cz) with 95% CIs during the presentation of notes in the familiar and unfamiliar melody conditions. The middle panel shows the mean amplitude of the N1 calculated from the interval of 79–99 ms (peak latency was 89 ms). The right panel shows the topographic distribution of the N1 (79–99 ms).
